# Mixed Diethanolamine and Polyethyleneimine with Enhanced CO_2_ Capture Capacity from Air

**DOI:** 10.1002/advs.202207253

**Published:** 2023-04-05

**Authors:** Yihe Miao, Yaozu Wang, Bingyao Ge, Zhijun He, Xuancan Zhu, Jia Li, Shanke Liu, Lijun Yu

**Affiliations:** ^1^ College of Smart Energy Shanghai Jiao Tong University No. 800 Dongchuan Road Shanghai 200240 China; ^2^ China‐UK Low Carbon College Shanghai Jiao Tong University No. 3 Yinlian Road Shanghai 201306 China; ^3^ Research Center of Solar Power & Refrigeration School of Mechanical Engineering Shanghai Jiao Tong University No. 800 Dongchuan Road Shanghai 200240 China; ^4^ The Hong Kong University of Science and Technology (Guangzhou) No.2 Huan Shi Road South Guangzhou Nansha, 511458 China; ^5^ Jiangmen Laboratory for Carbon and Climate Science and Technology No. 29 Jinzhou Road Jiangmen 529100 China

**Keywords:** diethanolamine, direct air capture, polyethyleneimine, sub‐ambient temperatures, supported mixed amine adsorbents

## Abstract

Supported polyethyleneimine (PEI) adsorbent is one of the most promising commercial direct air capture (DAC) adsorbents with a long research history since 2002. Although great efforts have been input, there are still limited improvements for this material in its CO_2_ capacity and adsorption kinetics under ultradilute conditions. Supported PEI also suffers significantly reduced adsorption capacities when working at sub‐ambient temperatures. This study reports that mixing diethanolamine (DEA) into supported PEI can increase 46% and 176% of pseudoequilibrium CO_2_ capacities at DAC conditions compared to the supported PEI and DEA, respectively. The mixed DEA/PEI functionalized adsorbents maintain the adsorption capacity at sub‐ambient temperatures of −5 to 25 °C. In comparison, a 55% reduction of CO_2_ capacity is observed for supported PEI when the operating temperature decreases from 25 to −5 °C. In addition, the supported mixed DEA/PEI with a ratio of 1:1 also shows fast desorption kinetics at temperatures as low as 70 °C, resulting in maintaining high thermal and chemical stability over 50 DAC cycles with a high average CO_2_ working capacity of 1.29 mmol g^−1^. These findings suggest that the concept of “mixed amine”, widely studied in the solvent system, is also practical to supported amine for DAC applications.

## Graphical abstract

Diethanolamine (DEA) incorporation is used to enhance the performance of supported polyethyleneimine (PEI) adsorbent for direct air capture. Supported mixed DEA/PEI‐functionalized adsorbents exhibit up to 46% enhanced CO_2_ capacity, over 100% improved amine efficiency, excellent adsorption at sub‐ambient temperatures, fast desorption kinetics at 70 °C, and high thermal and chemical stability over multiple cycles, compared to the single PEI‐loaded sample.

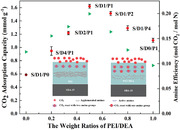

## 1 Introduction

Negative emission technologies (NETs) are acknowledged to play an essential role in achieving net zero emissions to tackle global warming.^[^ ^1^ ^]^ Compared to bioenergy with carbon capture and storage and afforestation with the uncertainty of land requirement and reduction emission potential, direct air capture (DAC) is becoming one of the most environmentally benign choices for NETs.^[^ ^1c,d^ ^]^ DAC systems absorb/adsorb atmospheric air in ambient conditions and regenerate and concentrate CO_2_ at high temperatures. Amine‐functionalized mesoporous materials, also named supported amine, are promising CO_2_ chemisorption adsorbents for DAC systems due to their high adsorption capacity, fast adsorption kinetics, and relatively low regeneration energy consumption.^[^ ^2^ ^]^ Supported amine materials can be categorized into three classes of adsorbents, including Class 1 (physical impregnation), Class 2 (covalent tethering via silane linkage), and Class 3 (covalent tethering via in situ polymerization).^[^ ^2^, ^3^ ^]^ Class 1 adsorbents attract most research interests since they balance low fabrication cost and excellent adsorption performance well.

Understanding the adsorption mechanism of supported amine is essential to improve adsorbents' performance further. According to the in situ infrared spectroscopy (IR) characterization, a reaction mechanism of polyamine‐impregnated adsorbents was proposed by the Chuang group in which primary amine sites react with CO_2_ forming a strongly bound ammonium carbamate, and secondary amine sites adsorb CO_2_ comprising a weakly bound carbamic acid.^[^ ^4^ ^]^ The surface species produced by the CO_2_ adsorption on the grafted primary amine (3‐aminopropyltrimethoxysilane)and secondary amine (N‐methyl‐3‐aminopropyltrimethoxysilane) are also varied.^[^ ^5^ ^]^ Accordingly, the theoretical amine efficiency, referring to the maximum CO_2_/N ratio of the chemical adsorption, is 0.5 for supported amine adsorbents in dry conditions.^[^ ^6^ ^]^ However, the experimental amine efficiency is usually far below 0.5 when capturing CO_2_ from air.^[^ ^2^, ^6^, ^7^ ^]^ The low amine efficiency can be ascribed to the intrinsic viscosity and aggregation of polyamine that inhibit the access of CO_2_ to active amine sites.^[^ ^8^ ^]^ Chuang's work suggested that when the CO_2_ concentration was below 1%, CO_2_ was not likely to react with the secondary amine sites in tetraethylenepentamine (TEPA), which are less accessible to the neighboring zwitterion to accept the proton.^[^ ^9^ ^]^

The additive of hydroxy‐rich poly(ethylene glycol) (PEG) has been verified to be able to improve the amine efficiency of polyethyleneimine (PEI) and TEPA‐impregnated composites.^[^ ^6^, ^7^, ^8^, ^10^ ^]^ Song group proposed in the presence of hydroxyl groups, CO_2_ can react with one single amine group so that the amine efficiency of PEI‐modified adsorbents can be improved.^[^ ^10^, ^11^ ^]^ Chuang group concluded that the introduced hydroxyl groups of PEG form hydrogen bonds toward ‐NH_2_ groups of TEPA polyamine, improve the dispersion of polyamine on supports, then decrease the CO_2_ diffusion resistance.^[^ ^6^, ^10^ ^]^ Jones group identified the optimal PEG/PEI ratio to reach the maximum amine efficiency is equal to the equimolar OH/reactive (1°, 2°) amine ratios.^[^ ^7a^ ^]^ They also suggested that the addition of PEG can reduce the cluster of PEI, demoting the CO_2_‐induced cross‐linking of amines. Wang et. al.^[^ ^8^ ^]^ proposed PEG not only accelerates CO_2_ diffusion but also stabilizes the adsorbed CO_2_ at low temperatures by changing the CO_2_‐amine speciation from carbamate to carbamic acid. However, the total CO_2_ capacity of adsorbents is negatively affected by the replacement of inert PEG.^[^ ^12^ ^]^ Additionally, the effects of inducing hydroxyl groups on the adsorption kinetics and cyclic stability of amine‐impregnated DAC adsorbents at low operating temperatures have not been investigated.

The supported mixed amine adsorbents were reported to be able to improve adsorption performance.^[^ ^7^, ^13^ ^]^ Diethanolamine (DEA) is widely reported to achieve the enhancement of both CO_2_ capacity and amine efficiency for supported TEPA adsorbents to capture pure CO_2_. For instance, SBA‐15 loaded with 30 wt.% TEPA and 20 wt.% DEA showed a CO_2_ adsorption capacity of 3.70 mmol g^−1^ at 75 °C and 100 kPa of CO_2_, larger than SBA‐15 loaded with the same amount of TEPA (3.27 mmol g^−1^) and DEA (0.47 mmol g^−1^).^[^ ^13a^ ^]^ Yogo group concluded 40 wt.% TEPA and 30 wt.% DEA modified mesostructured cellular form silica (MSU‐F) exhibited an enhanced CO_2_ capacity of 5.91 mmol g^−1^ at 50 °C capturing pure CO_2_, compared to 4.17 mmol g^−1^ of 70 wt.% TEPA‐MSU‐F.^[^ ^13b,c,e^ ^]^ These works suggest the hydroxyl groups of DEA cannot only increase the accessibility between CO_2_ and amines but also stabilize the carbamate anion through hydrogen bonding (R_1_R_2_NCOO^−^—HOR_3_). Unfortunately, our recent research work indicates the enhancement of mixed DEA/TEPA‐modified adsorbents is not significant under DAC conditions.^[^ ^7e^ ^]^ Therefore, the synergistic effect of mixed amine might be distinct under different CO_2_ concentrations.

Fast CO_2_ desorption at low regeneration temperature is also essential to cut down the operating cost of DAC systems. Introducing hydroxy‐rich additives can accelerate desorption by forming hydrogen bonds with nearby amine molecules to create CO_2_ pathways toward amine groups.^[^ ^8^, ^10^ ^]^ Choi group reported that 1,2‐epoxybutane functionalization on polyamines could reduce regeneration heat and improve thermal stability.^[^ ^14^ ^]^ The epoxide functionalization prompts the alkylation of polyamines with 2‐ethyl‐hydroxyethyl groups (–CH_2_CH(C_2_H_5_)OH), so the majority of primary amines are converted to secondary amines.^[^ ^14a^ ^]^ The heat of CO_2_ adsorption for supported amine can also be reduced by adding additives with 2‐hydroxybutyl groups, which reduce the basicity of the amine sites and provide a steric hindrance.^[^ ^14d,f^ ^]^ Another epoxide, propylene oxide, was blended into pentaethylenehexamineand TEPA with enhanced desorption performance.^[^ ^15^ ^]^ Note that the above strategies also cause the reduction of the CO_2_ capacity of adsorbents.

In this work, we show mixing DEA into PEI/SBA‐15 while fixing the total organic loading achieves unexpectedly improved CO_2_ adsorption and desorption performance for capturing CO_2_ from the air. The mixed amine of a ratio of 1:1 modified adsorbent exhibits a maximum pseudoquilibrium uptake of 1.62 mmol g^−1^, which is 45.9% higher than PEI/SBA‐15. Meanwhile, the supported mixed DEA/PEI composites display excellent adsorption performance at sub‐ambient temperatures. The introduced DEA also reduces the optimal regeneration temperature, so it lowers regeneration energy consumption and gains better long‐term cyclic stability.

## 2 Results and Discussion

### 2.1 Supported Mixed DEA/PEI Adsorbents Characterization

In one of our recent works, considering the balance between thermodynatics and kinetics of supported amine, 50 wt.% amine loading was examined to be the optimal loading ratio for polyamine‐impregnated SBA‐15 adsorbents for DAC.^[^ ^16^ ^]^ Thus, the total mixed amine loading was fixed at 50 wt.% in this work. The synthesized adsorbents are named as S/Dx/Py, where S represents SBA‐15, and x/y denotes the weight ratio of DEA(D) to PEI(P), respectively.

**Figure 1:**
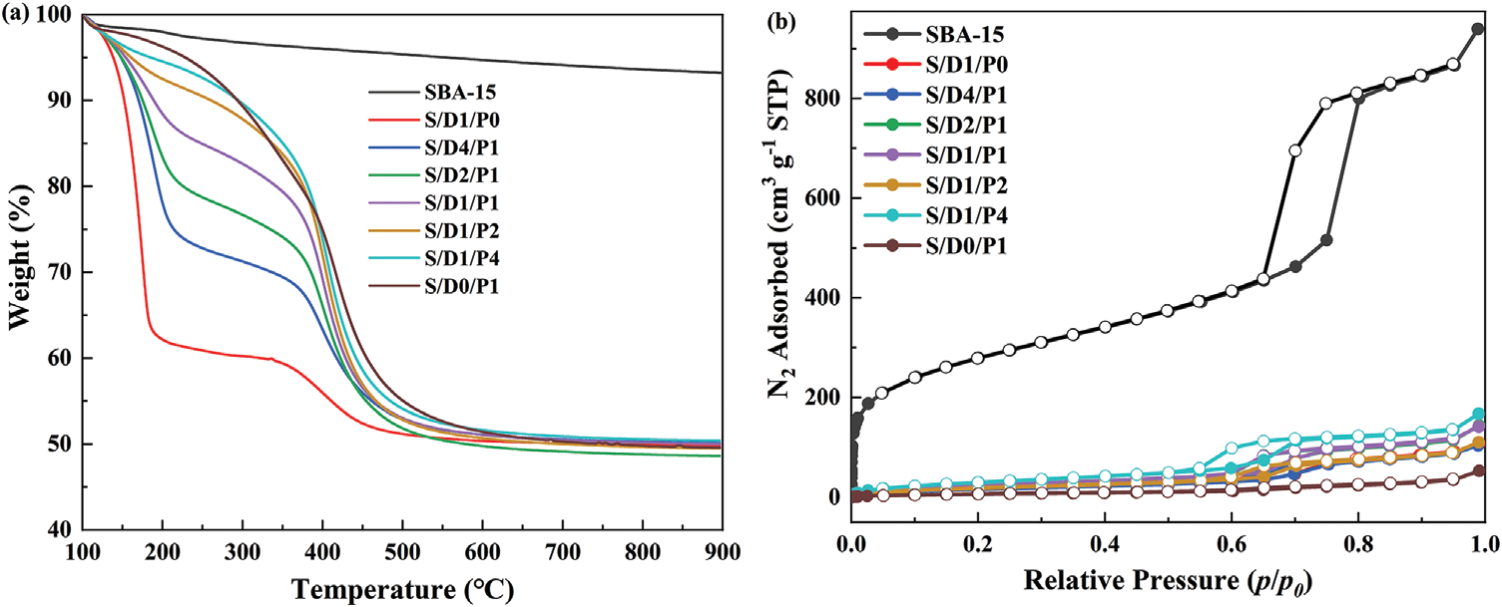
a) Thermal gravimetric analysis of supported mixed DEA/PEI with different blended ratios. b) N_2_ isotherms for adsorbents with different blended ratios of DEA and PEI. (Filled circles present the adsorption isotherm, and empty circles present the desorption isotherm, respectively.)

The results of TGA, shown in **Figure** **1**a, substantiate that the actual loading is very close to the set ratio of 50 wt.%, representing that the blended amines were impregnated on mesoporous substrates. As small molecular amine DEA is volatile, the sample of S/D1/P0 is thermally unstable compared to the polymer PEI‐loaded adsorbent (S/D0/P1). The thermal stability of mixed DEA/PEI adsorbents might be between a single DEA‐modified SBA‐15, and a single PEI functionalized SBA‐15, according to the TGA and DTG in Figure 1a and Figure S1 (Supporting Information).

The Fourier transform infrared spectrometer (FT‐IR) spectra in Figure S2 (Supporting Information) also verify that the blended amines were successfully loaded on SBA‐15. The vibration peak at 1560 cm^−1^, representing the primary amine group ‐NH_2_, appeared with the existence of PEI due to the DEA molecules only including the secondary amine group ‐NH.

The N_2_ isotherms, obtained at 77 K, of mixed amine‐modified adsorbents, are used to analyze the textural properties. A classic type IV adsorption‐desorption curve with the hysteresis loop, depicted in Figure 1b, presenting a sample with a mesoporous structure, can be observed for SBA‐15. Although DEA and PEI have a similar density, the samples with inducing DEA exhibit a larger pore volume compared to S/P1/D0 with minimal pore volume (Table S1, Supporting Information). It might result from the induing of DEA can decrease viscosity of PEI, therefore mitigating the agglomeration of polymer PEI inside SBA‐15.

### 2.2 Synergistic Adsorption Performance of Supported Mixed DEA/PEI Adsorbents

**Figure 2:**
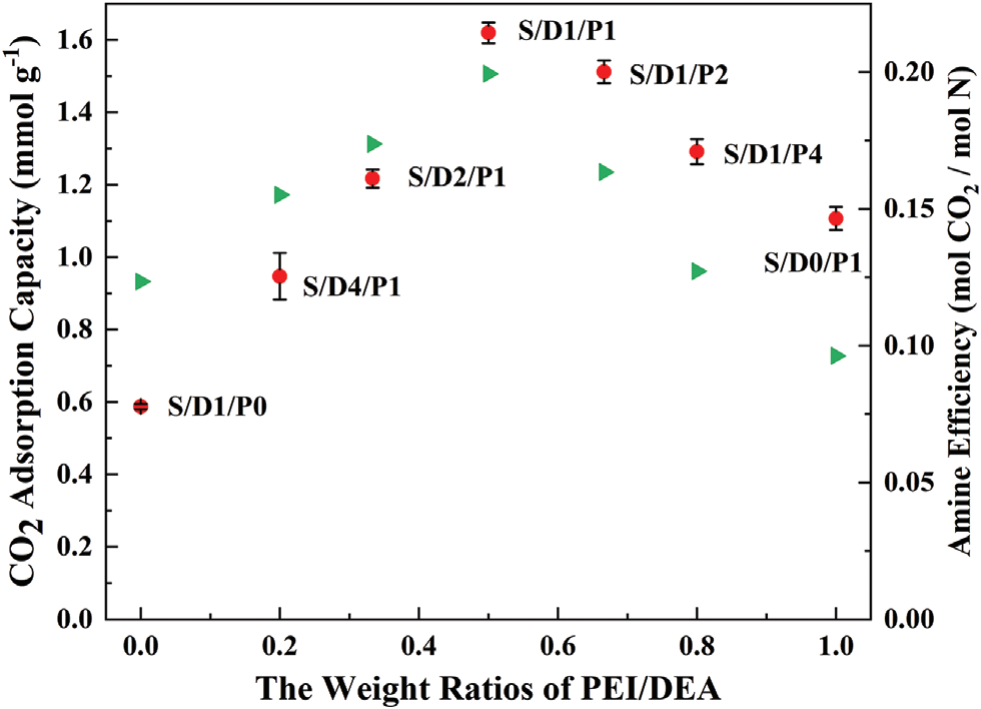
The CO_2_ adsorption capacities and amine efficiency of supported mixed amine adsorbents with different blended weight ratios of PEI and DEA obtained by TGA at 25 °C under the flow of 400 ppm CO_2_/N_2_.

The pseudoquilibrium CO_2_ adsorption capacities were gravimetrically measured by exposing samples to simulated air for 2 h after degassing pretreatments, as exhibited in **Figure** **2** and Figure S4 (Supporting Information). Interestingly, supported mixed DEA/PEI adsorbents reveal a significant synergistic effect on improving capacities. If no synergistic effect being considered, the resulting average capacities of supported mixed amine composites enhanced by 37%, 60%, 91%, 62%, and 29%, respectively, for the samples of S/D4/P1, S/D2/P1, S/D1/P1, S/D1/P2, and S/D1/P4, as concluded in **Table** **1**. As depicted in Figure S4b (Supporting Information), the appropriate introduction of DEA into PEI could significantly enlarge the adsorption period with high adsorption rates of adsorbents at 25 °C, resulting in improved CO_2_ capacities and amine efficiency. After around the first 60 min, the adsorption rate of samples with different blended ratios became similar and close to zero, probably representing the domination changing from rapid chemical adsorption to slow physical diffusion adsorption.

**Table 1:** CO_2_ adsorption capacity, the molar ratio of hydroxyl to amine, and amine efficiency at 25 °C

Samples	Capacity [mmol g^−1^]^a)^	“1 + 1 = 2” Capacity [mmol CO_2_ g^−1^] ^b)^	OH/N (molar ratio)	Amine efficiency [mol CO_2_/mol N]	OH/“Active” N (molar ratio)	Amine efficiency [mol CO_2_/mol “Active” N]
SBA‐15	0.104	∖	∖	∖	∖	∖
S/D1/P0	0.587	∖	2.00	0.12	2.00	0.12
S/D4/P1	0.947	0.691	1.25	0.16	1.44	0.18
S/D2/P1	1.217	0.760	0.91	0.17	1.13	0.22
S/D1/P1	1.620	0.847	0.59	0.20	0.79	0.27
S/D1/P2	1.512	0.934	0.34	0.16	0.49	0.23
S/D1/P4	1.291	1.003	0.19	0.13	0.28	0.19
S/D0/P1	1.107	∖	0	0.10	0	0.15

^a)^ The average of gravimetrical pseudoquilibrium capacities obtained by TGA under DAC conditions;

^b)^ “1 + 1 = 2” Capacity was calculated by simply using the blended ratio to obtain the value of capacities. For example, capacity of S/D1/P1 = 0587 * 0.5 + 1.107 * 0.5 = 0.847;

^c)^ “Active” N includes primary amine and secondary amine.

According to the literature, the positive effects of inducing hydroxyl groups into active amines for capturing CO_2_ can be ascribed to two principal reasons. From the perspective of reaction rate, the adsorption rate depends on the accessibility of CO_2_. The hydroxyl groups are likely to mitigate the agglomeration and CO_2_‐induced cross‐linking of polyamines, thus reducing the diffusion resistance of CO_2_ and enlarging the CO_2_ adsorption rate.^[^ ^7^, ^8^ ^]^ From the viewpoint of the reaction mechanism, the hydroxyl groups might change CO_2_ to react with one amine group instead of two amine groups by changing the CO2‐amine speciation from carbamate to carbamic acid.

**Figure 3:**
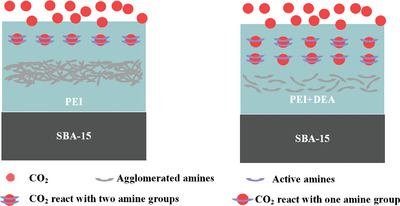
Schematic illustration of supported PEI and mixed DEA/PEI adsorbents capture CO_2_ from air.

In terms of mixed DEA/PEI modified adsorbents, though S/D0/P1 with the highest N loading, S/D2/P1, S/D1/P1, S/D1/P2, and S/D1/P4 exhibit comparably high initial momentary adsorption rate. That possibly results from the inducing hydroxyl groups decreasing the diffusion resistance of CO_2_. In addition, the reaction mechanism of supported mixed amine adsorbents with CO_2_ might change, thus contributing to the enlarged chemical dominant adsorption period and amine efficiency. The synergistic effect for mixed DEA/PEI composites under DAC condition, as illustrated in **Figure** **3**.

**Figure 4:**
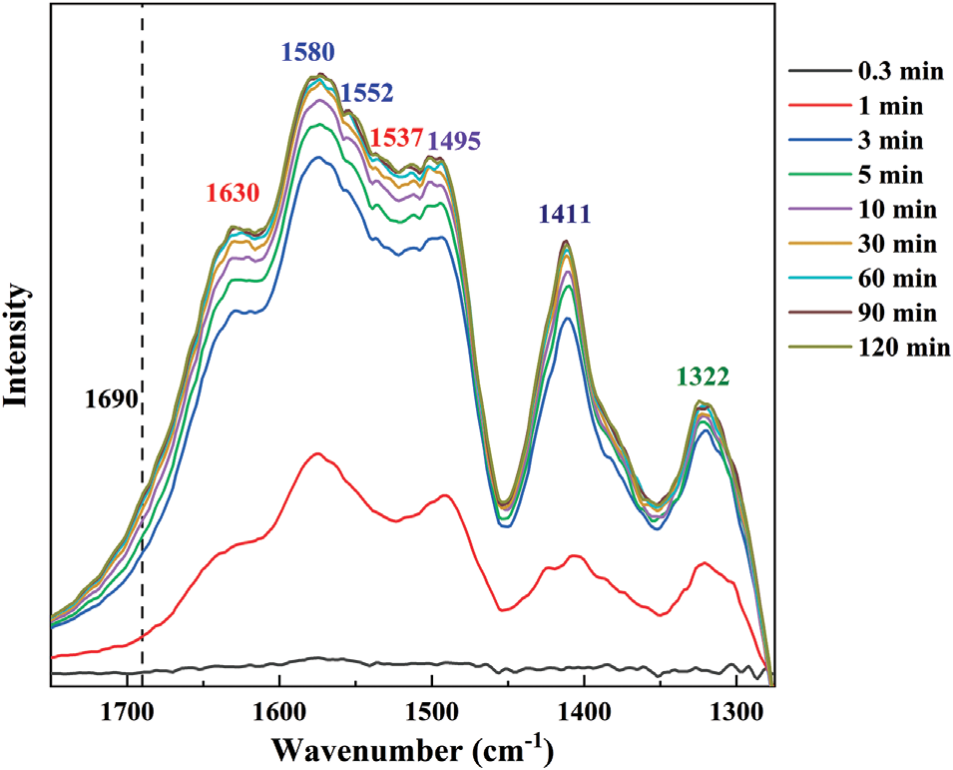
IR spectra for 400 ppm CO_2_/N_2_ adsorption on S/D1/P1 over 120 min.

The IR spectra for the adsorption of 400 ppm CO_2_/N_2_ over S/D1/P1 for 120 min are exhibited in **Figure** **4**. The shoulder at 1690 cm^−1^ is widely reported as solid evidence of the production of carbamic acid.^[^ ^4^, ^5^, ^9^, ^10^ ^]^ With the increasing adsorption time, an increase in the IR spectra at 1630, 1537, and 1411 cm^−1^, corresponding to the appearance of the specie of ammonium ion (NH_3_^+^, NH_2_^+^).^[^ ^4^, ^5^ ^]^ The peaks ≈1580 and 1552 cm^−1^ are assigned to the specie of carbamate ion (COO^−^ stretching).^[^ ^5^ ^]^ The intense peak at 1495 cm^−1^ is associated with carbamate ion (*δ*NH).^[^ ^5^ ^]^ The peak at 1322 cm^−1^ is attributed to NCOO^−^ skeletal vibration.

**Figure 5:**
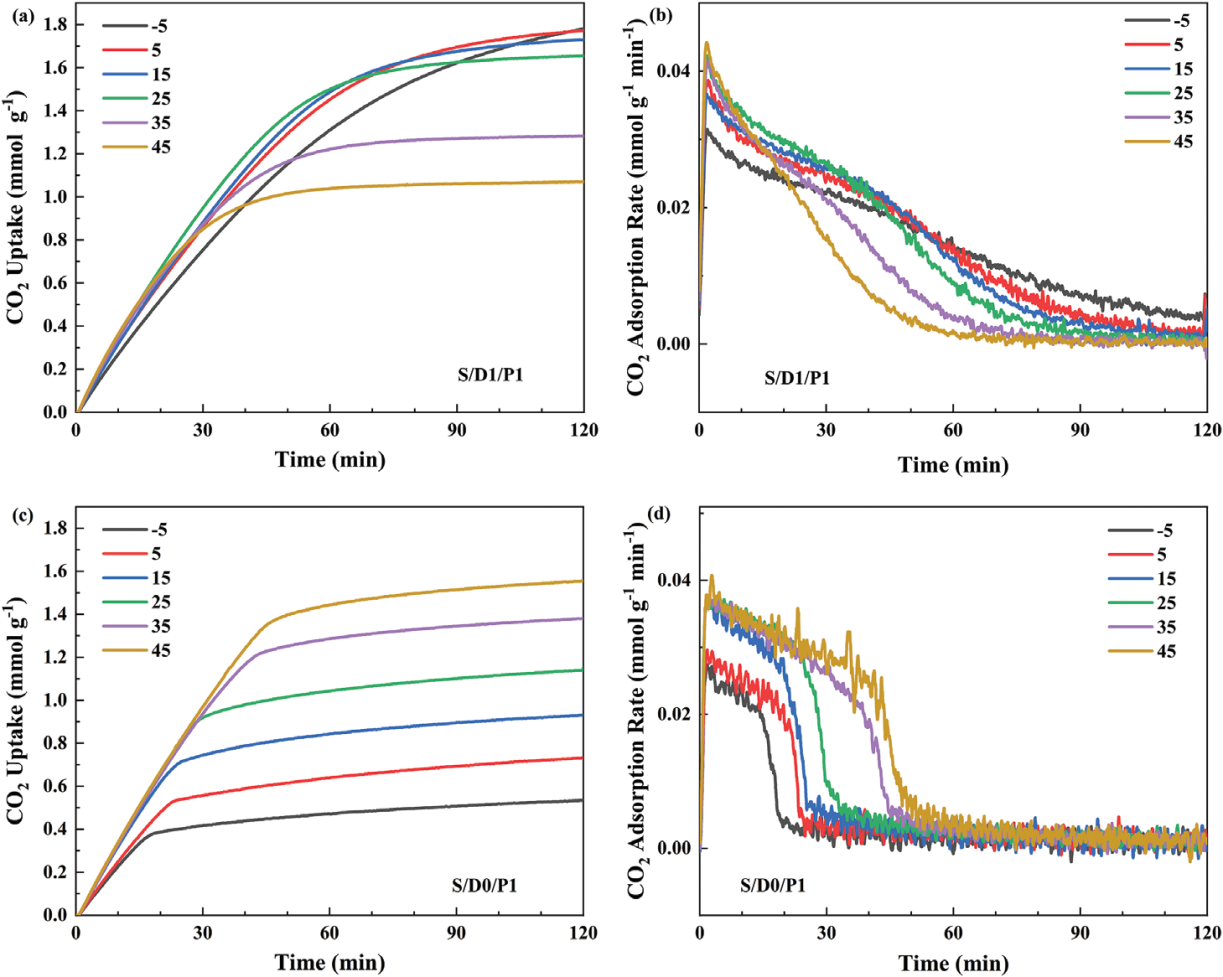
The comparison of the capacities of a) S/D1/P1 and c) S/D0/P1 at different adsorption temperatures. The corresponding CO_2_ adsorption rates of b) S/D1/P1 and d) S/D0/P1.

The performance of adsorbents at sub‐ambient temperatures is essential to actual DAC applications, while few studies conducted relative works.^[^ ^17^ ^]^ Our previous research has reported that the optimal adsorption temperature of PEI‐impregnated SBA‐15 is 45 °C, and the adsorption capacity and the adsorption rate reduce dramatically with the decreasing adsorption temperature.^[^ ^16^, ^18^ ^]^ The adsorption performance of supported PEI, as one of the most promising DAC adsorbents, highly depends on the operating temperature, which is likely to impede their applications in actual DAC conditions. This work examined the effect of adsorption temperatures on the performance of the supported mixed DEA/PEI adsorbents. Surprisingly, with the introduction of DEA, the supported mixed amine adsorbents exhibited different adsorption behaviors compared to the PEI/SBA‐15 composite, especially at sub‐ambient temperatures, as displayed in **Figure** **5** and Figure S5 (Supporting Information).

Although the adsorption rate of supported mixed DEA/PEI slightly reduces with the decreasing operating temperatures (Figure 5b), the mixed DEA/PEI‐impregnated SBA‐15 composites show excellent adsorption capacities at sub‐ambient temperatures. The reducing CO_2_ diffusion resistance among polyaminesand the change of reaction mechnism likely contributeto this outstanding adsorption performance after introducing hydroxyl groups. In particular, the sample of S/D2/P1, in contrast to S/D0/P1, even exhibits escalated uptakes with the reducing operating temperatures, representing that the thermodynamics is gradually becoming a capital factor for the adsorption process after introducing DEA. Among those supported mixed amine adsorbents, the adsorption performance of S/D1/P1 displayed less dependence on the operating temperatures and outstanding adsorption uptakes even at sub‐ambient temperatures.

### 2.3 Enhanced Desorption Kinetics of Supported Mixed DEA/PEI Adsorbents

**Figure 6:**
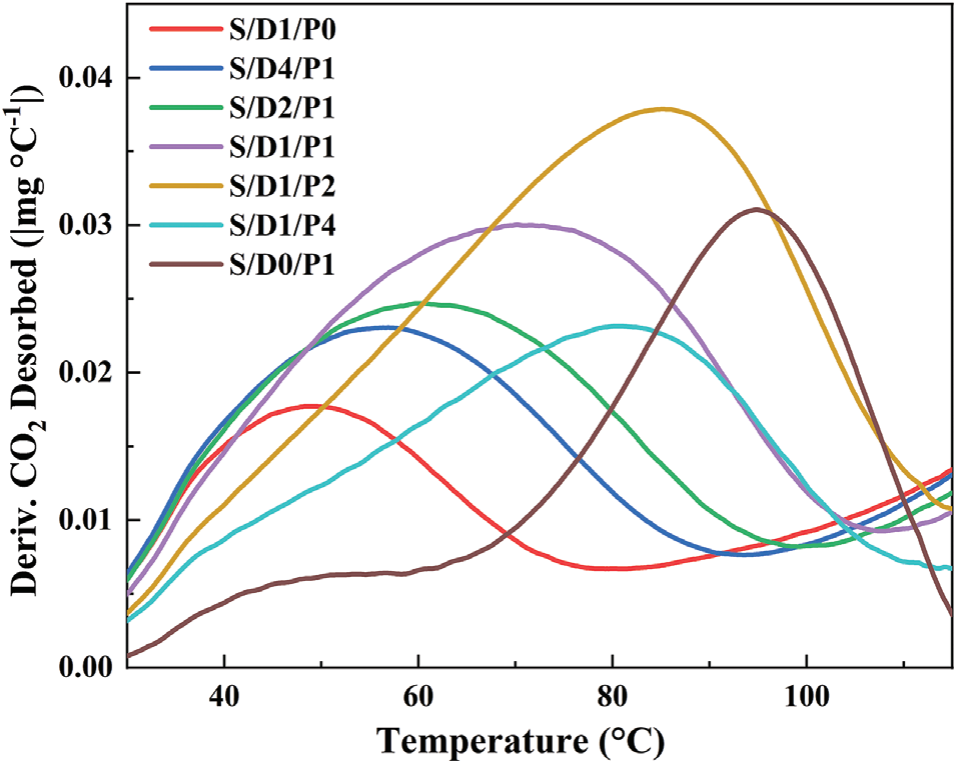
Temperature‐programmed desorption profiles for supported DEA/PEI adsorbents.

The desorption operating temperature not only necessitates the energy consumption of the regeneration process but also plays a vital role in conserving adsorbents' thermal and oxidative stability.^[^ ^18^, ^19^ ^]^ One of our recent works has reported that 90°C is an optimal regeneration temperature for S/D0/P1 to completely degas adsorbed CO_2_ in a short time, such as 10–15 min.^[^ ^16^ ^]^ This work examined the different degassing temperatures to ascertain the optimal regeneration temperature for mixed amine functionalized adsorbents, as displayed in Figure S7 (Supporting Information). It is interesting to note that the introduction of DEA can significantly decline the optimal regeneration temperature. That is likely ascribed to the reduced basicity of the amine sites after blending DEA into PEI weakened the interaction with CO_2_, revealed by the temperature‐programmed desorption results in **Figure** **6**.

At optimal regeneration temperature, the CO_2_ desorption rate, represented by the derivative of weight loss, should be close to zero after the rapid degassing stage to ensure complete degassing and avoid further loaded amine loss at high temperatures. For samples of S/D1/P1 and S/D1/P2, the optimal regeneration temperature reduces to 80 °C. S/D2/P1 could even achieve complete regeneration near 70 °C. The CO_2_ desorption processes for supported amine adsorbents degassing at 80 °C were normalized to compare the desorption kinetics, as shown in Figure S8 (Supporting Information). The desorption time is shorter for supported mixed DEA/PEI composites compared to single PEI‐impregnated SBA‐15. The sample S/D1/P0 likely suffers from decomposing at 80 °C for a long‐time, causing its highest 90% desorption time after normalization among these samples.

In addition, the Avrami model was used to fit the desorption processes of these supported mixed DEA/PEI adsorbents, as concluded in Figure S9 (Supporting Information) and Table S3 (Supporting Information). The fitting accuracy decreases with the increasing operation degassing temperatures. The higher degassing temperature likely caused the loss of impregnated amines, which can be confirmed by the gradually increased weight loss of adsorbents when the degassing temperatures were above the optimal regeneration temperature.

Given the fast adsorption and desorption stages completed in 60 and 15 min, respectively, as depicted in Figures S4,S8 (Supporting Information), only the first 60 min of adsorption and 15 min desorption processes were taken into consideration for cyclic stability tests. Herein, we designated the capacity adsorbed in 60 min at 25 °C in the cyclic test as the working capacity to compare the cyclic working performance of these supported mixed DEA/PEI adsorbents. As expected, S/D1/P1 exhibited excellent cyclic performance with an average working capacity of 1.40 mmol g^−1^ and a slight capacity loss after 10 cycles of 3.44% when the regeneration temperature was 80 °C. Meanwhile, S/D1/P1 also exhibited comparable cyclic performance under humid condition (Figure S11, Supporting Information).

**Figure 7:**
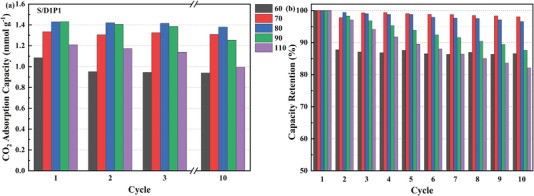
a) The change of CO_2_ adsorption capacities of the first, second, third, and 10th cycles over 10 cycles during 10 adsorption/ desorption cycles for S/D1/P1. b) The normalized cycle capacities of adsorbents during 10 adsorption/ desorption cycles for S/D1/P1. Adsorption was carried out under 400 ppm CO_2_ in N_2_ at 25 °C.

Although the capacity loss after 10 cycles of S/D0/P1 is 3.04% degassing at its optimal temperature of 90 °C, the average working capacity of S/D0/P1 is only 1.03 mmol CO_2_ g^−1^, which is ≈73% of that of S/D1/P1, as shown in Figure S10 (Supporting Information). If the degassing temperature decreases to 80 °C, the average working capacity of S/D0/P1 is only 0.91 mmol CO_2_ g^−1^, which is only ≈64.5% of that of S/D1/P1. Furthermore, the capacity loss after 10 cycles of these supported mixed amine adsorbents regenerating at their optimal degassing temperature is similar, performing comparable cyclic stability to the benchmark sample of S/D0/P1, as displayed in **Figure** **7**.

The working capacity of S/D2/P1 is higher than that of S/D1/P4 at low degassing temperatures, such as 70 and 80 °C, due to its lower optimal regeneration temperature, even though the pseudoquilibrium capacities of S/D1/P4 are higher at 25 °C (Figure 2). In addition, since the regeneration cannot complete at low regeneration temperatures, such as 60 °C, in 15 min, the first working capacity of these supported mixed DEA/PEI adsorbents is larger than that of the other nine cycles at 60 °C.

### 2.4 Anti‐Oxidative Stability and Long‐Term Stability

**Figure 8:**
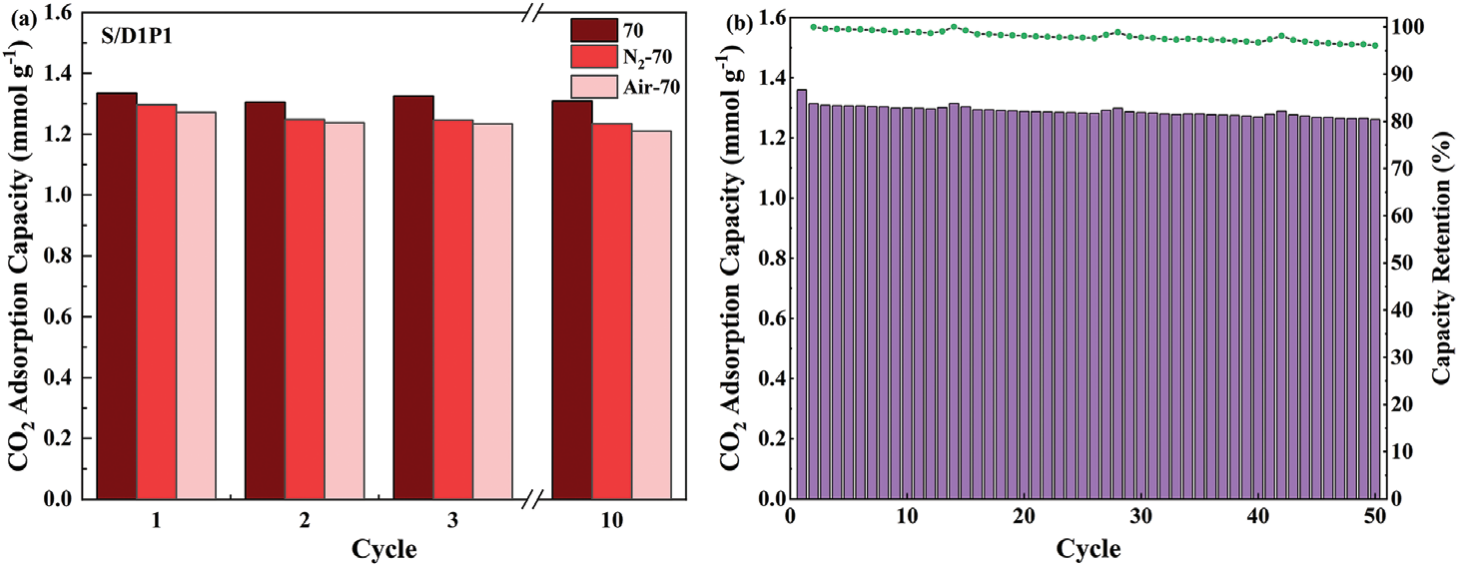
a) The cyclic CO_2_ adsorption capacities for S/D1/P1 composites degassing at 70 °C with different treatment conditions. The corresponding samples after 24 h thermal and oxidative treatments were denoted as N_2_‐70 and Air‐70, respectively. b) The long‐term working stability test of 50 cycles of S/D1/P1. Adsorption was carried out under 400 ppm CO_2_ in N_2_ at 25 °C for all samples. The regeneration temperature is 70 °C for S/D1/P1 according to the anti‐oxidation strategy.

Given the superior performance of adsorption and desorption kinetics, S/D1/P1 is assessed as the most promising candidate among these supported mixed DEA/PEI adsorbents. The accelerated thermal and oxidative stability tests of S/D1/P1 were then carried out, as exhibited in **Figure** **8**a and Figure S12 (Supporting Information). According to the anti‐oxidation strategy proposed in our previous work,^[^ ^18^ ^]^ the ideal anti‐oxidation degassing temperature should be slightly lower than the optimal regeneration temperature obtained only from the aspect of desorption kinetics. Usually, 10 °C is suggested, such as 70 °C in terms of S/D1/P1. Figure 8a, Figure S13 (Supporting Information), and **Table** **2** concluded that S/D1/P1 displayed corresponding thermal and oxidative stability to S/D0/P1 at their ideal anti‐oxidation degassing temperatures, even though the thermal stability of supported mixed amine is lower than S/D0/P1 (Figure 1a). Air‐assisted temperature swing adsorption for S/D1/P1 was also examined (Figure S14, Supporting Information), and S/D1/P1 exhibits nearly twice the working capacities of S/D0/P1.

**Table 2:** The cyclic average capacity of S/D1/P1 and S/D0/P1 without/with accelerated thermal or oxidative treatment at different operating temperatures

	Average working capacity of 10 cycles [mmol g^−1^]		
	Ideal cyclic test	After 24 h exposure to N_2_	After 24 h exposure to air
S/D1/P1‐70^a)^	1.3168	1.2408 (94.23%)	1.2244 (92.98%)
S/D0/P1‐70^a)^, ^b)^	0.7157	0.6937 (96.93%)	0.6712 (93.78%)
S/D0/P1‐80^a)^, ^b)^	0.9972	0.9619 (96.67%)	0.9211 (92.57%)

^a)^ The average capacity of the last 9 cycles excluding the first cycle;

^b)^ Data were cited from reference.^[^ ^18^ ^]^.

The 50‐cycle test was conducted, instructed by the anti‐oxidation strategy, to examine the long‐term working stability as shown in Figure 8b and Figure S15 (Supporting Information). The sample of S/D1/P1 can maintain an average working capacity of 1.29 mmol CO_2_ g^−1^ over the 50 cycles. After 50‐cycle working, S/D1/P1 and S/D0/P1 can retain over 96% of the initial working capacity. To our best knowledge,^[^ ^20^ ^]^ S/D1/P1 exhibited excellent long‐term stability among reported supported polyamine adsorbents for direct air capture.

## 3 Conclusion

The “mixed amine” concept was applied to supported amine adsorbents in this work to mitigate the aggregation of polyamine. The silica‐supported mixed DEA/PEI with a ratio of 1:1 exhibits the maximum CO_2_ uptake of 1.62 mmol CO_2_ g^−1^, which is 46% higher than supported PEI with the same amine loading. The outstanding adsorption performance of supported mixed amine can be maintained even at a cold temperature of −5 °C. In addition, the reduced basicity of supported mixed DEA/PEI composites leads to a decrease in desorption temperature to complete the regeneration process.

Under a reduced desorption temperature of 70 °C, the supported mixed DEA/PEI with a ratio of 1:1 displays comparable long‐term working stability to the supported PEI adsorbent during 50 adsorption‐desorption cycles. Considering its cheap raw materials and easy fabrication method, the supported mixed DEA/PEI adsorbent is a promising candidate for DAC applications.

## 4 Experimental Section

### Samples Preparation

SBA‐15 was synthesized by the templating method according to a previously reported procedure.^[^ ^7^, ^16^, ^21^ ^]^ The well‐prepared white SBA‐15 was stored in a brown container in ambient lab conditions. Mixed amine modified SBA‐15 sorbents were prepared by wet impregnation, followed by the previously published procedure.^[^ ^7^, ^16^ ^]^ In brief, the desired amount (total 200 mg) of the mixture of poly(ethylenimine) (PEI) (average Mw ≈800 by LS, average Mn ≈600 by GPC, Sigma‐Aldrich) and Diethanolamine [DEA, >99.0% (GC), TCI] (weight ratio = 1/4, 1/2, 1/1, 2/ 1, 4/1), respectively, was dissolved in methanol (10 mL, AR, Macklin) and stirred (800 rpm for 1 h). The overnight dried SBA‐15 (200 mg) was then added and stirred for a consecutive 6 h. After that, methanol was removed via rotary evaporation at room temperature, and the resulting amine‐modified composites were dried overnight at room temperature under vacuum (< 20 mTorr). These dried composites were stored in ambient lab conditions before use. The amine loading ratio was fixed as 50 wt.% for all samples. The synthesized adsorbents were named S/Dx/Py, where S represents SBA‐15, x/y denotes the weight ratio of DEA(D) to PEI(P), respectively.

### Characterization

Nitrogen physisorption isotherms of adsorbents were collected by a BELSORP‐MAX at 77 K. Before testing, the samples were subjected to vacuum (<20 mTorr) for degassing (at least 6 h at 60 °C). The total surface area was calculated by the Brunauer‐Emmett‐Teller theory through the collected adsorption points in the relative pressure range of 0.05 to 0.3, and the mesopore volume and mesopore size calculations were calculated by the non‐local density functional theorymodel. The thermal analysis was conducted by PerkinElmer TGA 8000. During the experiment, the sample was heated from room temperature to 900 °C (10 °C min^−1^) under nitrogen (99.9995%, Air Liquide) purging (100 mL min^−1^). The morphology of samples was obtained by using a scanning electron microscope (Nova NanoSEM 450) operating at 10 kV. Infrared spectra (IR) of the samples were collected between 4000 and 500 cm^−1^ by using Nicolet iS50 FTIR spectrometer (Thermo Fisher Scientific, USA). Quantitative element analysis of N, C, and H contents was performed using a Vario EL Cube elemental analyzer.

### Adsorption/Desorption Studies

Gravimetrical pseudoequilibrium CO_2_ adsorption capacities experiments were measured by a PerkinElmer TGA 8000. Estimations of CO_2_ capacities were attained by pretreating the given sample (≈5 mg) under N_2_ flow (100 mL min^−1^) at 90 °C for 1 h, followed by decreasing to 25 °C or the setting operating temperature (5 °C min^−1^), and then thermal equilibration for 30 min, and subsequently exposed to a flow of simulated air (400 ppm CO_2_ in N_2_, 100 mL min^−1^, Air Liquide) for 2 h. The adsorption at sub‐ambient temperatures was also conducted on TGA, and the detailed procedure was described elsewhere.^[^ ^16^, ^18^ ^]^ For the temperature programmed desorption (TPD) test, the CO_2_‐saturated samples were heated in pure N_2_ form from 25 to 120 °C at a heating rate of 5 °C min^−1^. The in situ IR DRIFTS experiment was conducted by an IR spectrometer (VERTEX 80v). The DRFITS cell was filled with solid sorbent (≈100 mg) and placed inside the spectrometer. The adsorption gaseous setup condition was the same as that of pseudoequilibrium CO_2_ adsorption capacities experiments. The IR signal was recorded three times per minute for the first 10 min, twice per minute until the 60 min, and every 1 min for the last 60 min during the adsorption with 32 scans at a resolution of 4 cm^−1^.

Cyclic adsorption/desorption experiments were simplified for ten adsorption/desorption cycles by adopting 15 min for degassing at a set temperature, 15 min for thermal equilibration under N_2_ flow, and 60 min for adsorption. For the first cycle of the cyclic test, the pretreating at a setting degassing temperature for 2 h to desorb the adsorbed water and CO_2_ completely. The accelerated thermal and oxidative treatments were conducted on TGA, and the detailed procedures were described elsewhere.^[^ ^18^ ^]^ The TGA was modified with an air‐free water bubbler to conduct the cyclic stability under humid condition at room temperature (25 °C) with a relative humidity of 20%. The humid N_2_ was induced after the 2 h pretreating process.

Volumetric pseudoequilibrium CO_2_ capacities were measured at various sorption temperatures by using a BELSORP‐MAX. Before the isotherm collection, the samples were degassed under a dynamic vacuum (<5 mTorr) at 60 °C for at least 6 h. An equilibration interval of 60 s was used for CO_2_ partial pressures <1 kPa and 30 s for all other partial pressures.

## Conflict of Interest

The authors declare no conflict of interest.

## Supporting information

Supporting Information

## Data Availability

The data that support the findings of this study are available in the supplementary material of this article.
