# Supplementary material for: Mixed Diethanolamine and Polyethyleneimine with Enhanced CO2 Capture Capacity from Air
Source: Adv Sci (Weinh). 2023 Apr 5;10(16):2207253. doi: 10.1002/advs.202207253 (PMC10238199; doi:10.1002/advs.202207253)
Supplement: Supplementary file 1 — Supporting Information [file ADVS-10-2207253-s001.pdf]

ADVANCED SCIENCE
Open Access

Supporting Information

for *Adv. Sci.*, DOI 10.1002/advs.202207253

Mixed Diethanolamine and Polyethyleneimine with Enhanced CO_2 Capture Capacity from Air

Yihe Miao, Yaozu Wang, Bingyao Ge, Zhijun He, Xuancan Zhu, Jia Li*, Shanke Liu and Lijun Yu**

Supporting Information

Mixed Diethanolamine and Polyethyleneimine with Enhanced CO_2 Capture Capacity from Air

Yihe Miao, Yaozu Wang, Bingyao Ge, Zhijun He, Xuancan Zhu, Jia Li*, Shanke Liu, Lijun Yu**

Table S1. The textural properties of adsorbents with different DEA and PEI blended ratios. The mesopore volume and mesopore size calculations were calculated by the non-local density functional theory (NLDFT) model.

Adsorbent	BET Specific surface area (m^2 g^{-1})	Pore volume (cm^3 g^{-1})
SBA-15	992	1.319
S/D1/P0	65	0.137
S/D4/P1	62	0.134
S/D2/P1	88	0.174
S/D1/P1	92	0.178
S/D1/P2	72	0.135
S/D1/P4	114	0.204
S/D0/P1	27	0.055

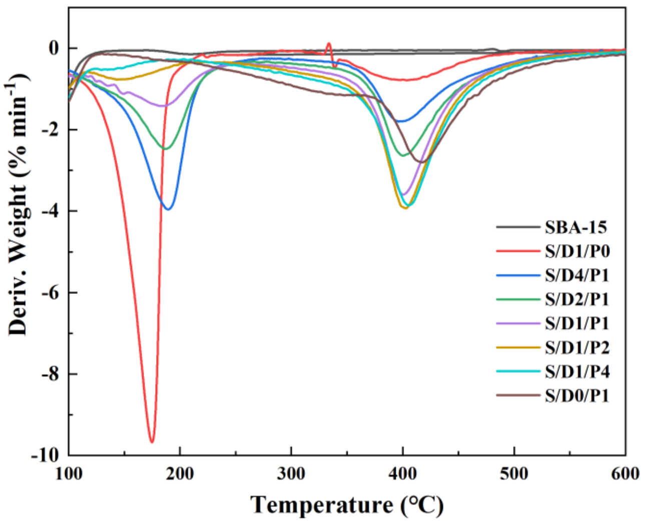

Figure S1. Derivative thermal gravimetric analysis of mixed DEA/PEI composites.

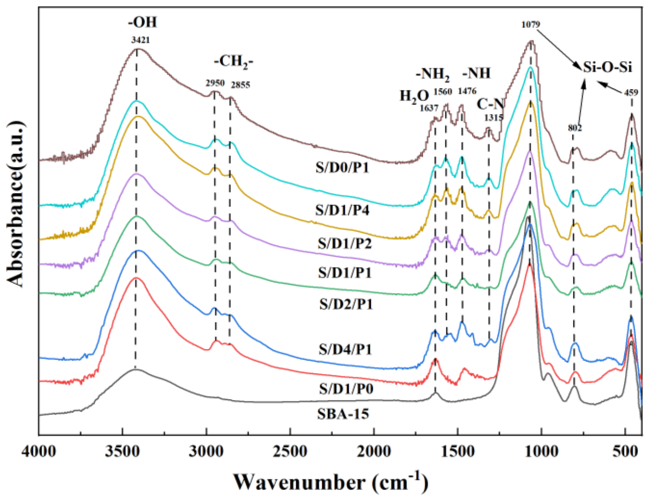

Figure S2. FT-IR spectra of mixed DEA/PEI modified adsorbents.

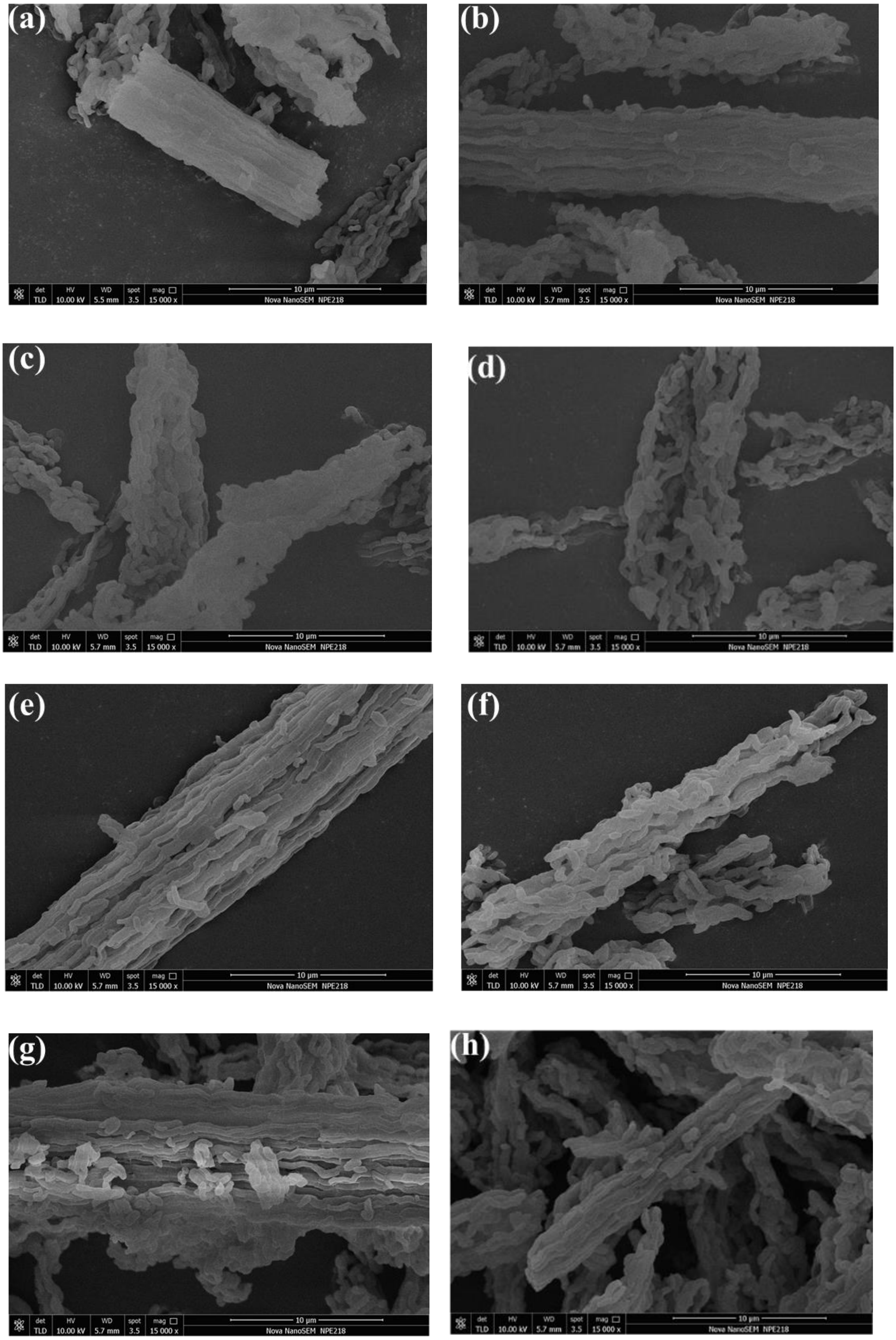

Figure S3. Scanning Electron Microscopy images of a) SBA-15, b) S/D1/P0, c) S/D4/P1, d) S/D2/P1, e) S/D1/P1, f) S/D1/P2, g) S/D1/P4, and h) S/D0/P1.

Table S2. The results of element analysis of supported amine adsorbents.

Adsorbent	N (%)	C (%)	H (%)
S/D1/P0	3.96	14.15	3.95
S/D2/P1	8.68	18.17	5.05
S/D1/P1	11.42	21.75	5.62
S/D1/P2	12.13	22.56	6.70
S/D0/P1	15.70	26.03	6.13

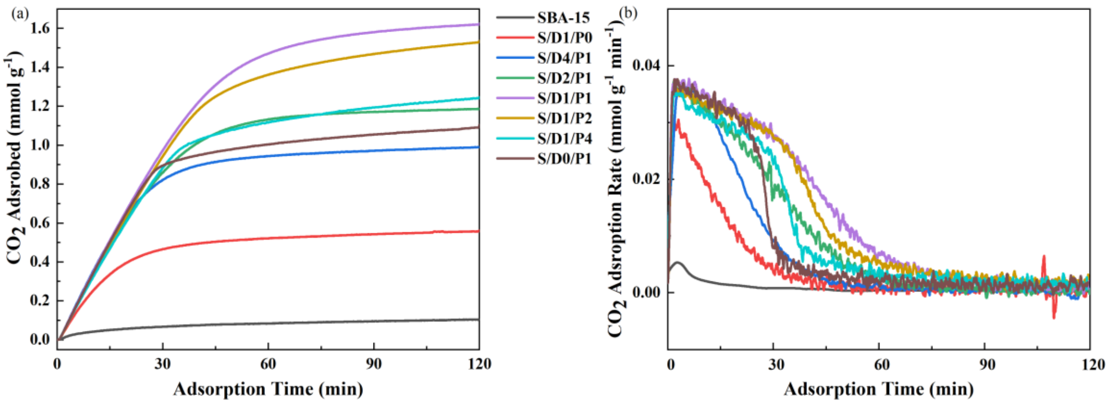

Figure S4. CO_2 adsorption kinetic tests for different weight ratios of PEI and DEA a) adsorption kinetics at 25 °C under the flow of 400 ppm CO_2/N_2, and b) the change of CO_2 adsorption rate.

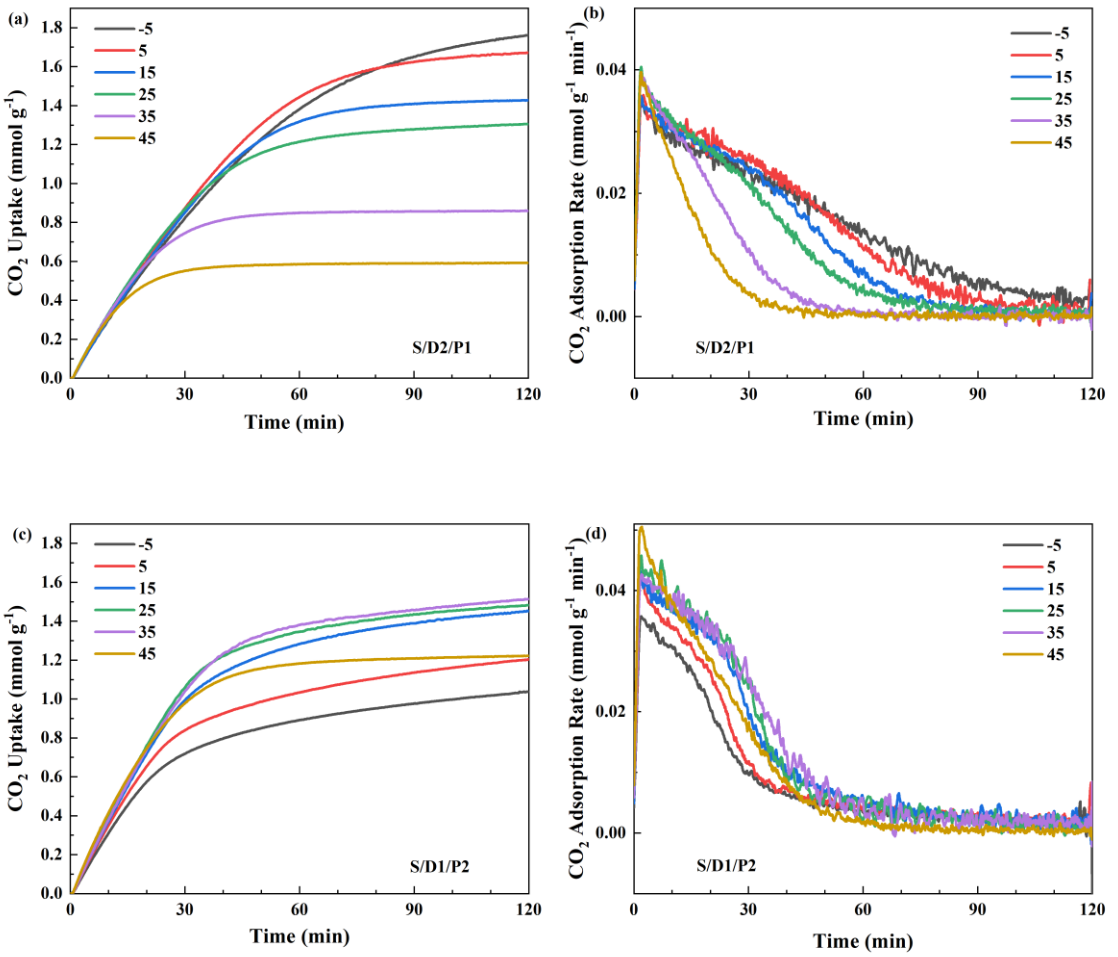

Figure S5. The comparison of the capacities of a) S/D2/P1 and c) S/D1/P2 at different adsorption temperatures. The corresponding CO_2 adsorption rates of b) S/D2/P1 and d) S/D1/P2.

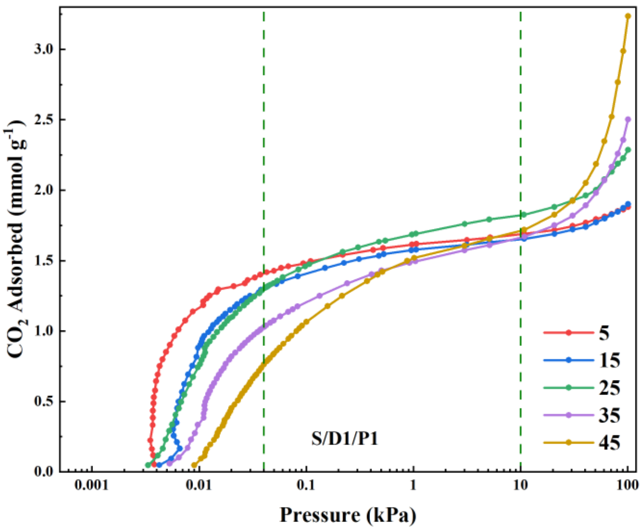

Figure S6. CO2 adsorption isotherms of S/D1/P1 at different operating temperatures.

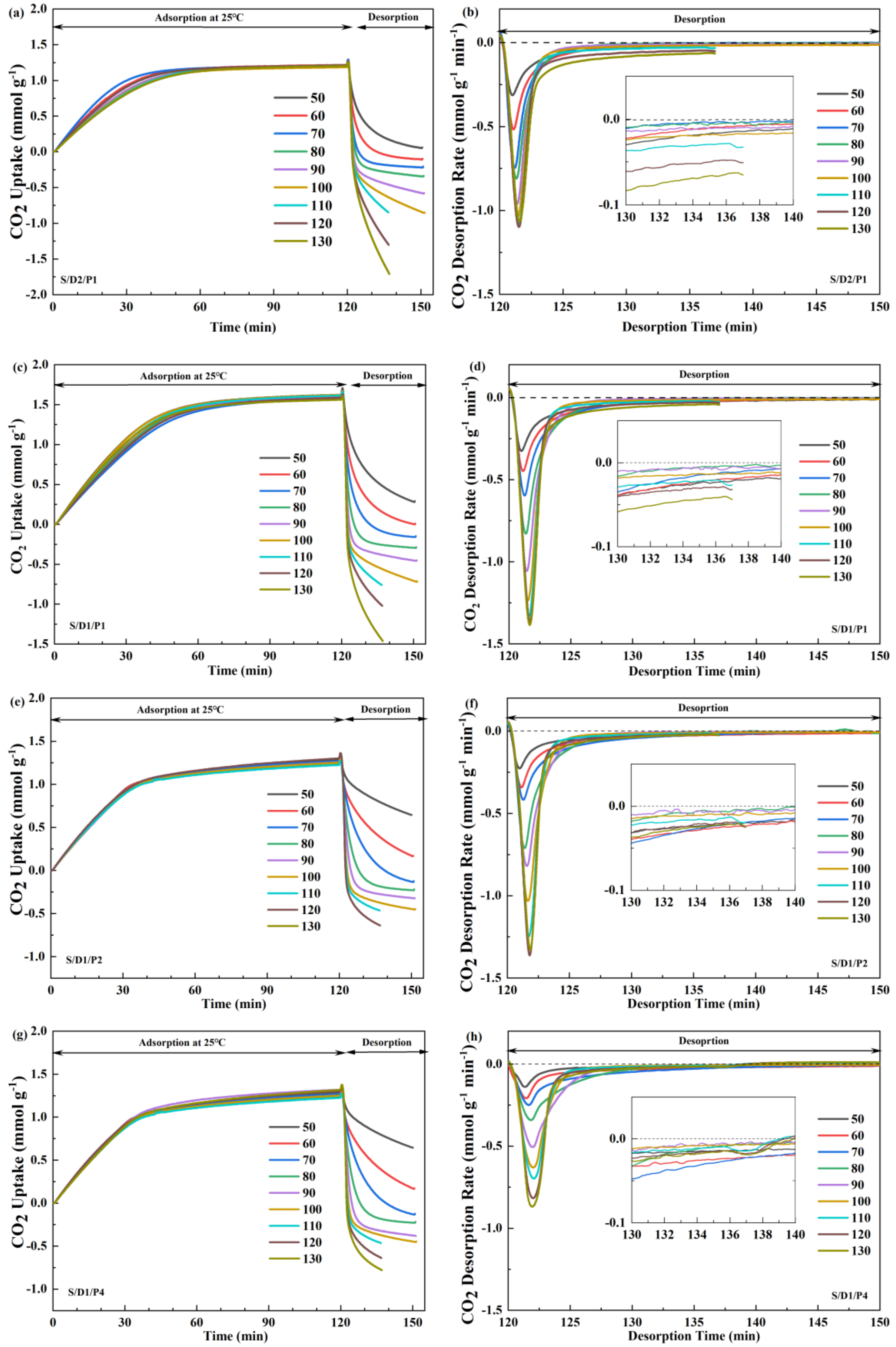

Figure S7. The profiles of CO_2 adsorption at 25 °C and desorption at different

degassing temperatures for a) S/D2/P1, c) S/D1/P1, e) S/D1/P2, and g) S/D1/P4. The desorption rate under a flow of N_2 at different temperatures for b) S/D2/P1, c) S/D1/P1, f) S/D1/P2, and h) S/D1/P4.

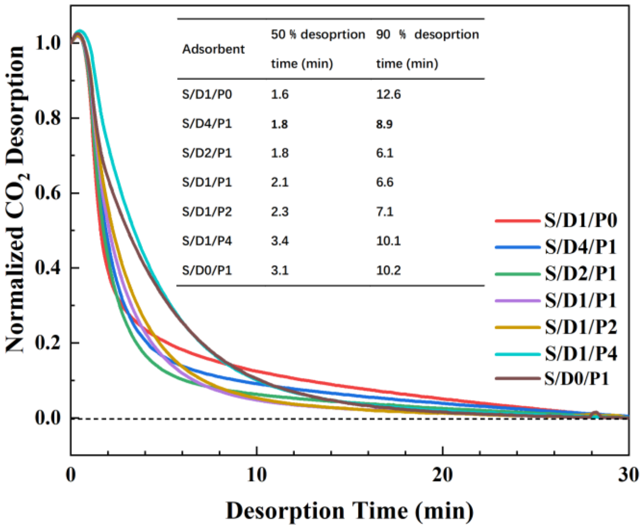

Figure S8. The normalized CO_2 desorption processes for supported DEA/PEI adsorbents degassing at 80 °C.

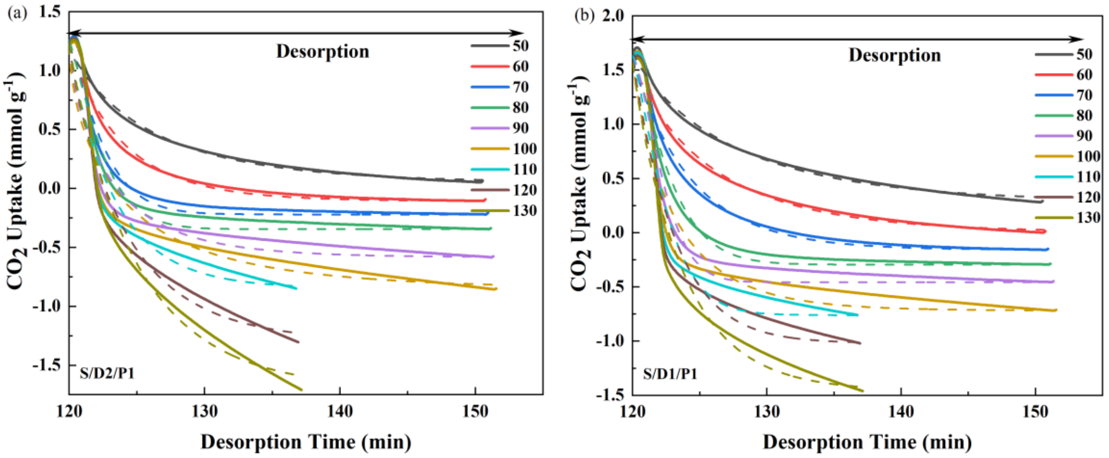

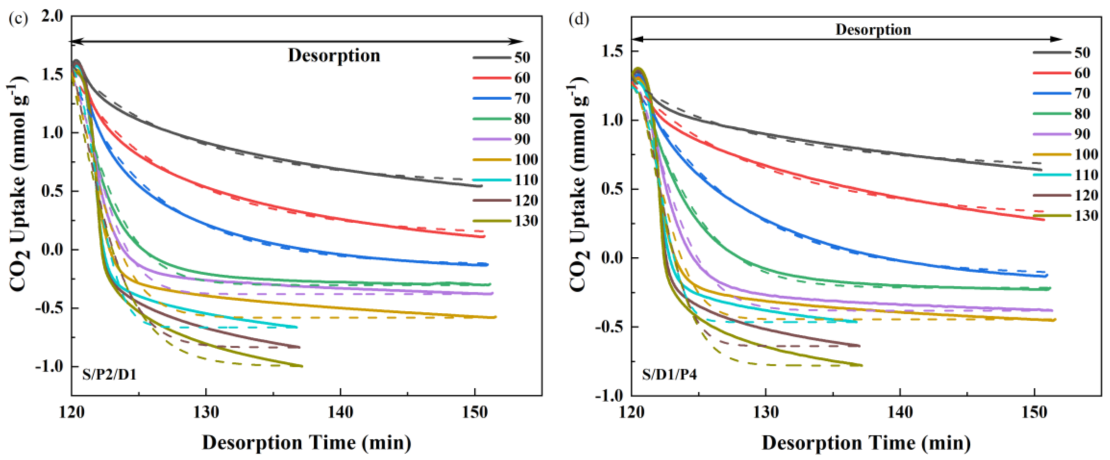

Figure S9. The desorption profile combined with corresponding fitting results through the Avrami model for a) S/D2/P1, b) S/D1/P1, c) S/D1/P2, and d) S/D1/P4 (solid lines: experimental data, dashed lines: Avrami model).

Table S3. Parameter values of the Avrami model of supported mixed amine for desorption at varying temperatures.

Adsorbent	Desorption temp. (°C)	Avrami model k	n	R^2	Err%
S/D2/P1	50	0.16209	0.85955	0.99084	6.96464E-2
	60	0.25371	0.92171	0.98183	0.163
	70	0.37134	1.21343	0.96993	0.275
	80	0.37306	1.25474	0.95147	0.508
	90	0.3269	0.77868	0.90149	1.26
	100	0.25219	0.6639	0.91071	1.546
	110	0.30031	0.94083	0.93283	1.917
	120	0.23993	0.89553	0.95355	1.977
	130	0.20146	0.9409	0.96856	1.88
S/D1/P1	50	0.13182	0.8783	0.99197	9.22666E-2
	60	0.1671	0.87675	0.9889	0.162
	70	0.22616	0.972	0.98471	0.252

	80	0.3202	1.26844	0.97877	0.377
	90	0.37924	1.49078	0.95267	0.888
	100	0.31948	0.83941	0.89393	2.428
	110	0.36544	1.27151	0.93069	2.754
	120	0.31475	1.04246	0.93089	3.273
	130	0.26892	0.96941	0.94634	3.383
S/D1/P2	50	0.10868	0.90666	1	7.08622E-2
	60	0.12817	0.88765	0.99105	0.112
	70	0.16066	0.94236	0.99131	0.142
	80	0.29342	1.26297	0.98178	0.296
	90	0.33002	1.4646	0.96084	0.642
	100	0.33685	1.29228	0.92135	1.543
	110	0.3907	1.82106	0.94729	1.918
	120	0.33763	1.28751	0.93176	2.876
	130	0.3028	1.17368	0.93938	2.777
S/D1/P4	50	0.10134	0.8568	0.97658	6.13037E-2
	60	0.09893	0.93313	0.98602	8.97535E-2
	70	0.12743	1.04185	0.99386	8.68164E-2
	80	0.20862	1.28242	0.99148	0.122
	90	0.26825	1.45901	0.97509	0.414
	100	0.32296	1.63917	0.94776	0.773
	110	0.35557	2.16908	0.96059	0.964
	120	0.33228	1.76699	0.94016	1.904
	130	0.31457	1.55828	0.93948	2.238

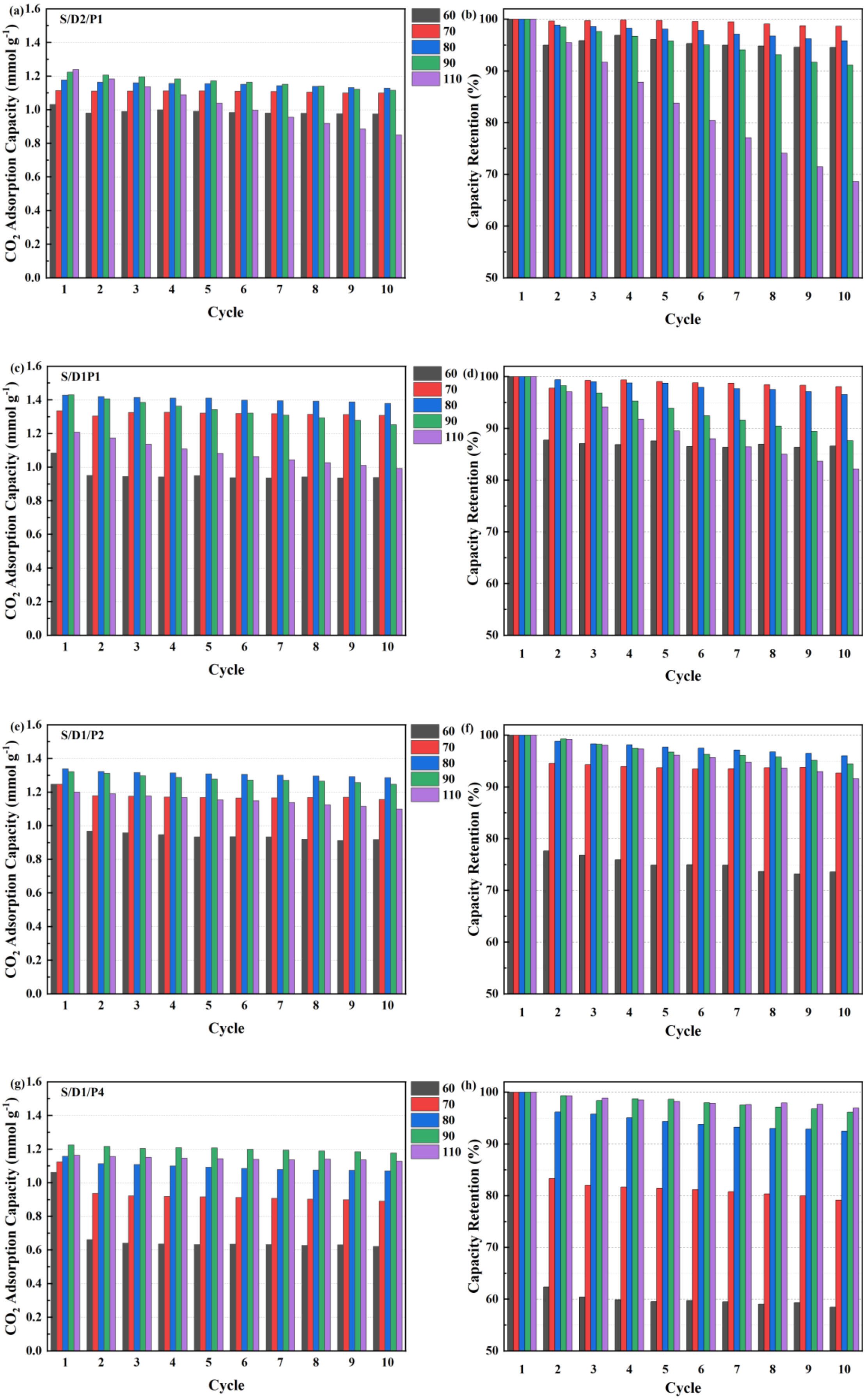
(a)
S/D2/P1
(b)
(c)
S/D1P1
(d)
(e)
S/D1/P2
(f)
(g)
S/D1/P4
(h)
60
70
80
90
110
CO_2 Adsorption Capacity (mmol g^{-1})
Capacity Retention (%)
Cycle

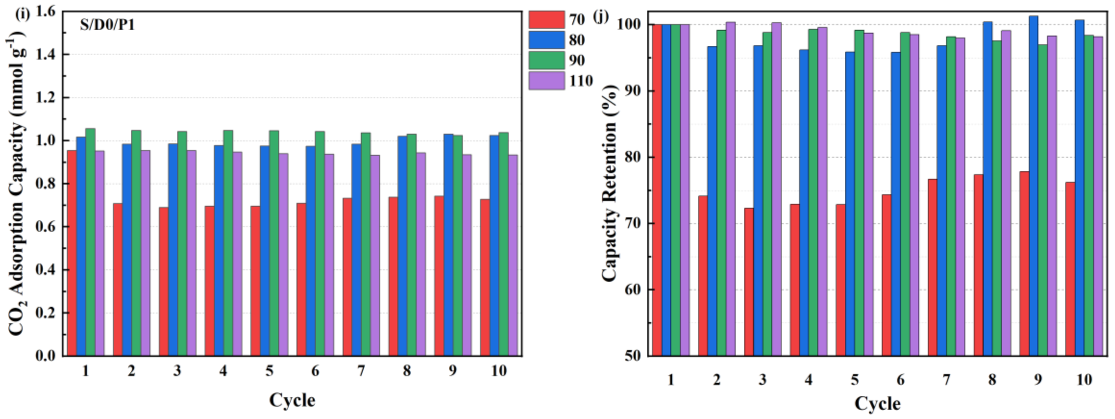

Figure S10 The change of CO_2 adsorption capacities and normalized cycle capacities of adsorbents during 10 adsorption/ desorption cycles for a, b) S/D2/P1, c, d) S/D1/P1, e, f) S/D1/P2, g, h) S/D1/P4 and i, j) S/D0/P1. Adsorption was carried out under 400 ppm CO_2 in N_2 at 25 °C for all samples. Data of S/D0/P1 were cited from Ref[2].

Table S4. The average working capacity of different supported mixed polyamines at different regeneration temperatures.

	Average working capacity of 10 cycles (mmol g^{-1})				
	60 °C	70 °C	80 °C	90 °C	110 °C
S/D2/P1	0.9840 [a]	1.1079 [a]	1.1510	1.1678	1.0297
S/D1/P1	0.9423 [a]	1.3168 [a]	1.4041	1.3385	1.0847
S/D1/P2	0.9363 [a]	1.1689 [a]	1.3085	1.2808	1.1518
S/D1/P4	0.6355 [a]	0.9123 [a]	1.0958	1.2010	1.1443
S/D0/P1 [b]	\	0.7157	0.9972	1.0413	0.9429

[a)] The average capacity of the last 9 cycles' values excluding the first cycle; [b)] Data for S/D0/P1 cited from Ref.[2]

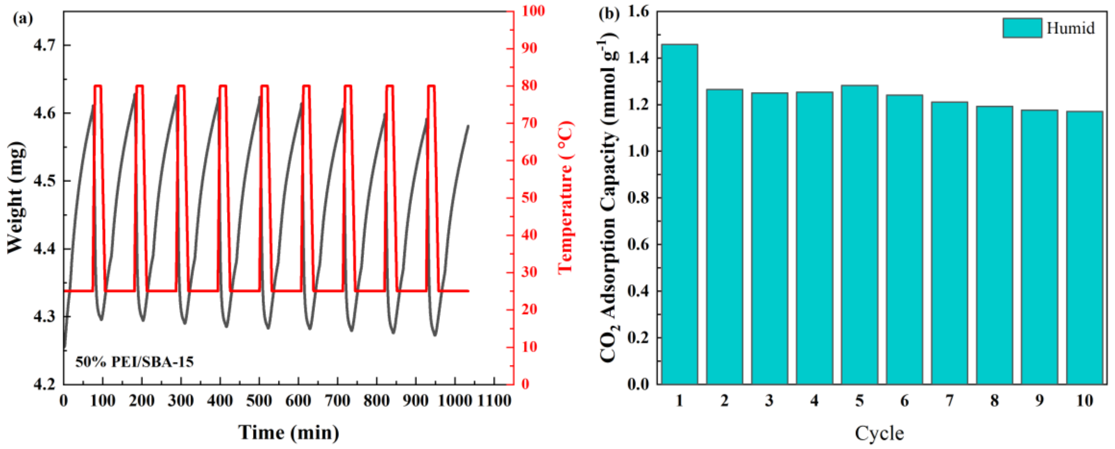

Figure S11. (a) CO_2 adsorption/desorption profile and (b) CO_2 cyclic capacities under humid condition (25 °C, 20% RH) for S/D1P1 over 10 cycles.

Table S5. The element analysis results of S/D1/P1 and S/D0/P1 without/with accelerated thermal and oxidative treatments.

Adsorbent	Pristine			After 24-h exposure to N_2			After 24-h exposure to air		
	N (%)	C (%)	H (%)	N (%)	C (%)	H (%)	N (%)	C (%)	H (%)
S/D1/P1-70	11.50	21.09	5.81	9.75	21.02	5.91	10.71	21.82	5.80
S/D0/P1-70	15.07	26.03	6.13	13.24	23.81	5.75	14.04	24.27	6.30
S/D0/P1-80	15.07	26.03	6.13	13.30	23.92	6.01	13.36	24.03	6.28

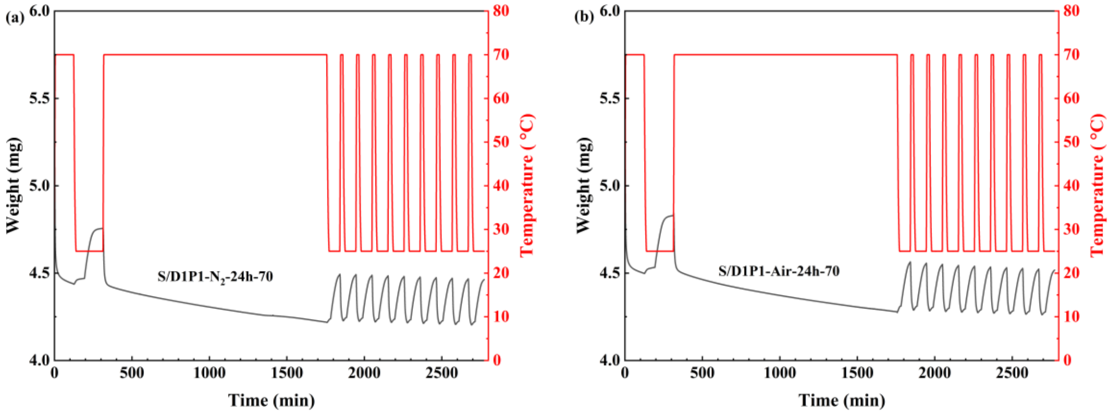

Figure S12. CO_2 adsorption/desorption profile for S/D1P1 degassing at 70 °C over 10 cycles after (a) 24-hour thermal treatment and (b) 24-hour oxidative treatment.

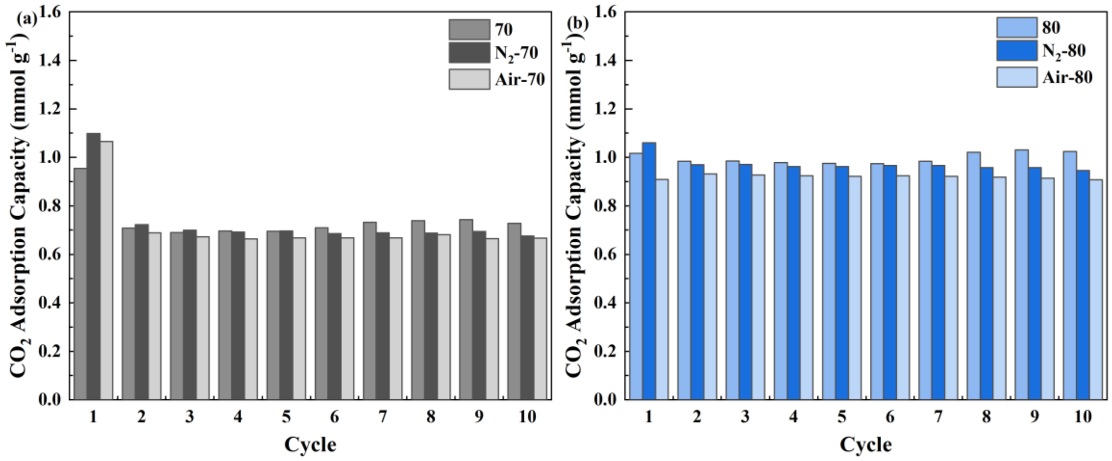

Figure S13. The cyclic CO_2 adsorption capacity for S/D0/P1 degassing at a) 70 °C and b) 80 °C with different treatment conditions. The corresponding samples after 24-h thermal and oxidative treatments were denoted as N2-70/80 and Air-70/80, respectively. Data were cited from Ref[2].

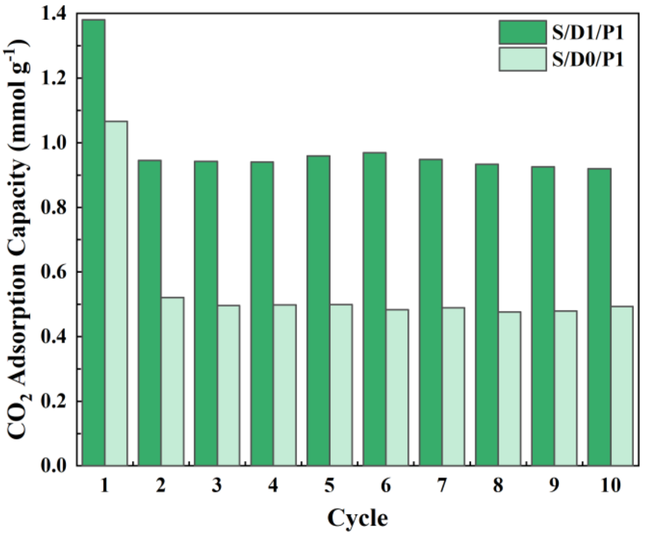

Figure S14. The comparison of cyclic CO2 adsorption capacities over 10 cycles of S/D1/P1 and S/D0/P1 at 70 °C and 80 °C, respectively. Adsorption was carried out under 400 ppm CO_2 in N_2 at 25 °C for all samples, and desorption was carried out under 400 ppm CO_2 in N_2 except the first cycle.

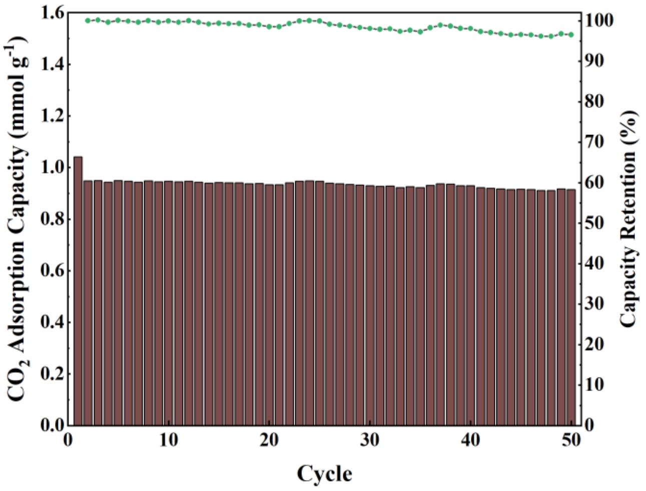

Figure S15. The long-term working stability test of 50 cycles of S/D0/P1. Adsorption was carried out under 400 ppm CO_2 in N_2 at 25 °C for all samples. The regeneration temperature is 80 °C for S/D0/P1 according to the anti-oxidation strategy.

Reference

[1] Z. He, Y. Wang, Y. Miao, H. Wang, X. Zhu, J. Li, Mixed polyamines promotes CO_2 adsorption from air, Journal of Environmental Chemical Engineering 10(2) (2022). https://doi.org/10.1016/j.jece.2022.107239.

[2] Y. Miao, Y. Wang, X. Zhu, W. Chen, Z. He, L. Yu, J. Li, Minimizing the effect of oxygen on supported polyamine for direct air capture, Sep. Purif. Technol. 298 (2022). https://doi.org/10.1016/j.seppur.2022.121583.
